# Consequence of a sudden wind event on the dynamics of a coastal phytoplankton community: an insight into specific population growth rates using a single cell high frequency approach

**DOI:** 10.3389/fmicb.2014.00485

**Published:** 2014-09-15

**Authors:** Mathilde Dugenne, Melilotus Thyssen, David Nerini, Claude Mante, Jean-Christophe Poggiale, Nicole Garcia, Fabrice Garcia, Gérald J. Grégori

**Affiliations:** Aix Marseille Université, Université de Toulon, CNRS/INSU, IRD, Mediterranean Institute of Oceanography, UM 110Marseille, France

**Keywords:** phytoplankton, flow cytometry, high frequency, *in situ* growth rates, Berre lagoon, wind

## Abstract

Phytoplankton is a key component in marine ecosystems. It is responsible for most of the marine primary production, particularly in eutrophic lagoons, where it frequently blooms. Because they are very sensitive to their environment, the dynamics of these microbial communities has to be observed over different time scales, however, assessment of short term variability is often out of reach of traditional monitoring methods. To overcome these limitations, we set up a Cytosense automated flow cytometer (Cytobuoy b.v.), designed for high frequency monitoring of phytoplankton composition, abundance, cell size, and pigment content, in one of the largest Mediterranean lagoons, the Berre lagoon (South-Eastern France). During October 2011, it recorded the cell optical properties of 12 groups of pico-, nano-, and microphytoplankton. Daily variations in the cluster optical properties were consistent with individual changes observed using microscopic imaging, during the cell cycle. We therefore used an adaptation of the size-structured matrix population model, developed by Sosik et al. (2003) to process the single cell analysis of the clusters and estimate the division rates of 2 dinoflagellate populations before, during, and after a strong wind event. The increase in the estimated *in situ* daily cluster growth rates suggest that physiological changes in the cells can prevail over the response of abundance.

## Introduction

Phytoplankton is responsible for about 50% of the annual global net primary production, yet its biomass represents only about 2% of the entire chlorophylous biomass (Field et al., 1998). This compartment is made up of mostly drifting autotrophic microorganisms, whose dynamics are important for carbon and nutrient fluxes in the ecosystem. With a high turnover compared to terrestrial plants (Margalef, 1978), phytoplankton plays an essential role in driving the biogeochemical cycles and the Redfield ratio in the ocean (Goldman et al., 1979). However, obtaining an accurate description of the dynamic distribution of phytoplankton can be limited by the methods available for measuring, on a short temporal scale, the processes which may influence it. Various methods have now been developed to investigate phytoplankton, from the single cell level (by microscopy and flow cytometry after *in situ* sampling), the global scale using bulk methods (*in situ* fluorimetry and metagenomics) up to the ocean scale using remote sensing. The ocean color obtained by satellite observations indicates bulk chlorophyll concentrations, used as a proxy for phytoplanktonic biomass, can be highly variable in coastal waters (Antoine et al., 1996). This variability is due to forcings, that control net population growth, grazing, and physical transport (Cloern, 1996), acting over hourly or daily scales. Since conventional sampling strategies hardly reach these scales, a legitimate concern has been raised over the underestimation of the natural short-term dynamics of phytoplankton during oceanographic campaigns and routine surveys (Rantajärvi et al., 1998). Or it is well known that the short generation time of phytoplankton can explain their ability to coexist on the same limited resources (Hutchinson, 1961).

The most efficient method for quantifying phytoplankton productivity is the estimation of the dividing rate of a population (in relation to the cell cycle) indicative of NPP. This combined with the variation in abundance is the only method for quantifying loss rates which is a good indicator of the sensitivity of the phytoplankton to trophic interactions or abiotic forcings. Estimations of dividing rates, expressed as population growth rate, are generally based on DNA measurements with lags from an hour to a day (Binder et al., 1996; Vaulot and Marie, 1999). Unfortunately, this requires important manual handling (i.e., sampling, fixing, storing, staining, and analysis) of the samples. To address these limitations, priority has been given to autonomous flow cytometry (Peeters et al., 1989; Olson et al., 2003), like the Cytosense flow cytometer (Cytobuoy b.v., Netherlands) in order to observe fine-scale temporal dynamics of phytoplankton (Dubelaar and Gerritzen, 2000; Dubelaar and Jonker, 2000; Dubelaar et al., 2004; Thyssen et al., 2008, 2014). Single cell analysis using several optical properties gives a good alternative for estimating growth rates, as they are linked to cell size. As previously reported in Durand (1995) and Binder et al. (1996), periodic increase and decrease in light scatter intensity can be interpreted as a response of cellular growth and division occurring during the cell cycle. Diel variations in the phytoplankton cell dimensions based on flow cytometry proxy therefore represent a necessary compromise for automated measurement requirements in order to calculate growth rates of natural phytoplanktonic groups during high frequency surveys (Sosik et al., 2003). Moreover, image processing allow to further improve the estimations of cell dimensions using mounted imaging systems implemented in the last generation of instruments (Sieracki et al., 1998).

In this study, we report on a high frequency *in situ* monitoring of phytoplankton in the Berre lagoon during October 2011. The Berre lagoon, located in South Eastern France is one of the largest brackish lagoons off the Mediterranean shore. Since 1995, the site has benefited from a special attention relying on a local ecological survey conducted by the GIPREB Joint Union (Groupement d'Intêret Public pour la Réhabilitation de l'Etang de Berre) to observe and report on the eutrophication of the lagoon. However, in this ecosystem, the influence of important seasonal events like natural floods or strong wind events, which may have an influence on light and nutrient availability, are poorly documented and may limit the full understanding of the mechanisms driving the phytoplanktonic variability (Gouze et al., 2008). If previous studies have reported a high eutrophic state characterized by the dominance of only a few species, more recent data highlights a high biodiversity, superior to that found in the nearby Bay of Marseille (Thyssen et al., 2011; Malkassian, 2012). Using a Cytosense automated flow cytometer, equipped with the “image-in-flow” device, abundances and optical properties were measured every hour for species ranging from pico- to small microphytoplankton. Pictures of microphytoplanktonic cells were used to estimate cell size from the sideward scatter signal and apply the full size distribution model developed by Sosik et al. (2003). *In situ* population growth rates were eventually determined and investigated with regards to the environmental variables monitored at the same frequency.

## Materials and methods

### Study area

The Berre lagoon is located on the SE French coast, close to the city of Marseille (800,000 inhabitants) and the Frioul archipelago where meteorological data (wind speed and direction) is permanently recorded by the meteorological station of the Mediterranean Institute of Oceanography (MIO). It is one of the largest brackish water pools off the Mediterranean shore (with a surface of 155 km^2^), receiving both seawater via the Caronte channel and freshwater inputs from natural and drifted tributaries (Arc, Touloubre and Durance rivers). It is divided into two shallow sub-basins: the “Grand Etang” (max. depth 9 m) and the “Etang de Vaïne” (max. depth 4 m) where the experiment took place. Every month, the GIPREB assesses the vertical structure of the water column at 10 hydrological stations, with one located in the “Etang de Vaïne.” Profiles of temperature, salinity, dissolved oxygen, pH, chlorophyll, turbidity and nutrients (*NO*^−^_3_, *NO*^−^_2_, *NH*^+^_4_, *PO*^3−^_4_) are measured at several depths to provide information on the stratification of the lagoon.

### Sampling system

**Figure 1:**
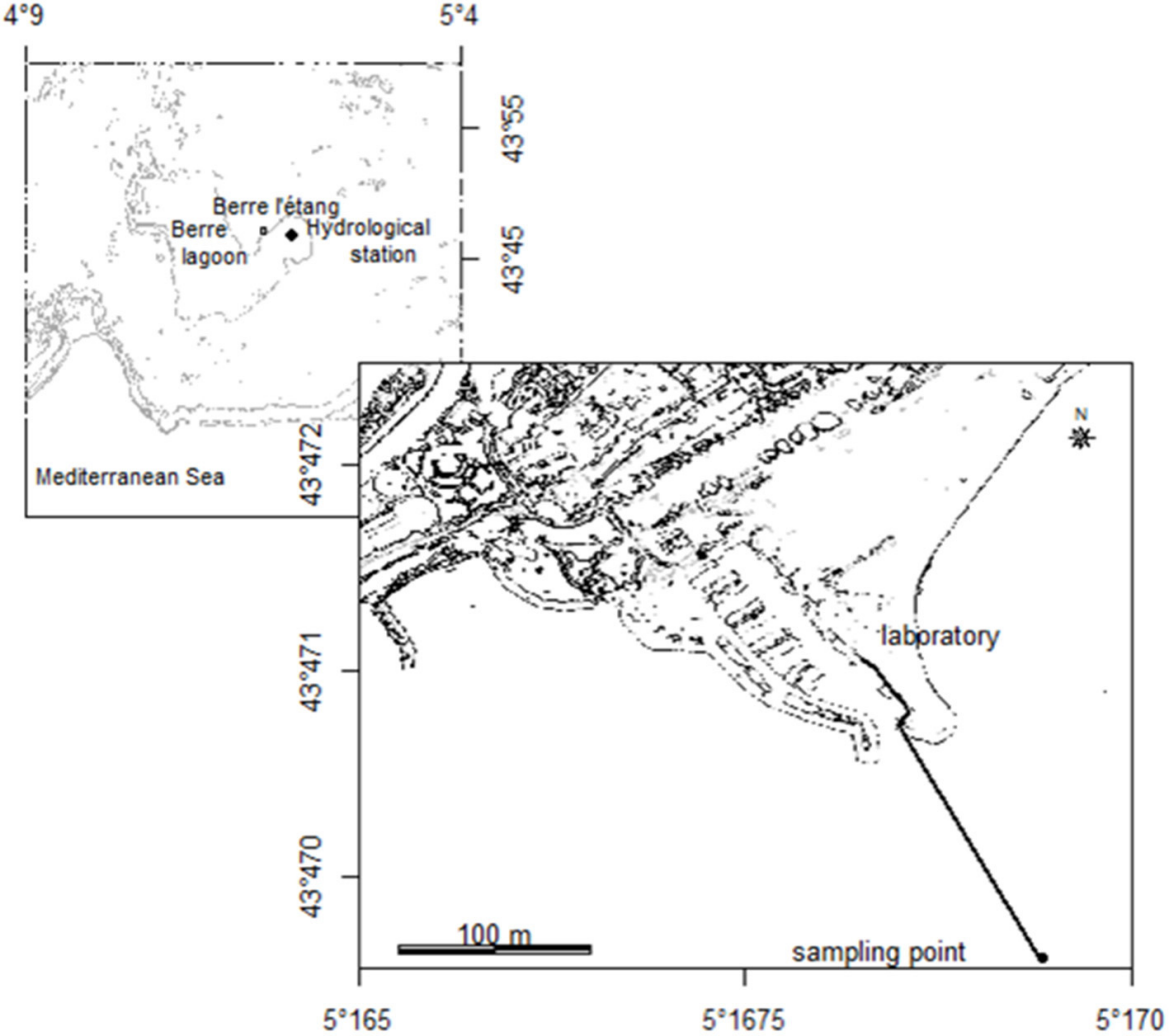
**Location of the sampling point (black circle) and the 250 m pipe (black line) to pump the water to the laboratory, settled on the Berre l'étang harbor**.

A multi-instrumental platform composed of a Cytosense automated flow cytometer and hydrological sensors was set up in the GIPREB laboratory located on the “Berre l'Etang” harbor (Figure 1). Water was pumped directly from the Vaïne lagoon (43°47 N, 5°17 E, 2.5 m depth) to the laboratory using a 250 m pipe (inner diameter 50 mm). Samples for flow cytometric analyses were taken every hour from a 1-dm^3^ sampling reservoir and hydrological sensors were placed in an 80-dm^3^ tank supplied after the subvolume. Every hour, water was pumped (JABASCO pump) at a flow rate of 30 dm^3^·min^−1^ for 17 min so that the pipe, the sampling reservoir and the tank were flushed and renewed several times prior to measurements. Samples were analyzed using the Cytosense 2 min after the pump stopped, when all air bubbles had disappeared. An evacuation pipe led the water back to the lagoon (by gravity). The JABASCO pump uses flexible impeller technology so that it does not squeeze the water passing through, therefore avoiding the damage to the cells (Thyssen et al., 2009).

Between October 1st and 14th 2011, nitrate (*NO*^−^_3_) concentration was measured using a Satlantic's ISUS V3 nitrate sensor set up in the 80-dm^3^ tank with a CTD (Hydrolab) which recorded temperature, chlorophyll concentration, turbidity, and salinity (conductivity) of the water. Throughout the month, the phytoplanktonic assemblage was characterized using the Cytosense of the PRECYM flow cytometry platform of the MIO (http://precym.mio.univ-amu.fr). Additional discrete samples were manually collected to determine nutrient (*NO*^−^_3_, *NO*^−^_2_, *PO*^3−^_4_) concentration and calibrate the ISUS sensor using a Technicon Autoanalyser (dectection limits: 50 nM for nitrate and 20 nM for nitrite and phosphate). Ammonium ions (*NH*^+^_4_) were measured by spectrophotometry. An *in situ* HOBO sensor, fixed near to the pipe inlet, measured incident light intensity and temperature.

### Flow cytometry

The Cytosense is a flow cytometer optimized to analyse phytoplankton. It has been specifically designed to analyse particles (cells, chains, colonies) of 1–800 μm diameter and up to several mm in length (chain forming cells). The sample flows in a sheath fluid to an exciting light source (a 488 nm laser beam) at a rate of 9 mm^3^·s^−1^ in order to optimize the processing of cells, separation, and alignment. The inherent optical properties of the cells were recorded in full pulse shape as they crossed the laser beam: sideward light scatter (SWS) and red (FLR, 668–734 nm), orange (FLO, 601–668 nm) and yellow (FLY, 536–601 nm) fluorescences were collected on photomultiplier tubes. Forward light scatter (FWS) was collected by a PIN photodiode. Data was acquired in Log scale. The cytoUSB software (Cytobuoy b.v.) was used to control the flow cytometer and acquire the data stored on a computer. Data acquisition was triggered on the chlorophyll-induced red fluorescence (FLR) of the phytoplankton cell. The threshold of the FLR signal was set at 9 mV in order to compute optical pulse shapes of the photosynthetic cells and get rid of the background noise (heterotrophs and detritic particles). Samples were hydrodynamically focused in the flow cytometer by the sheath fluid: just prior to the experiment, the cytometer was filled with 1 μm filtered seawater preserved with a 1% formaldehyde solution. After each analysis, the sheath fluid and the samples were mixed together and the sheath fluid was recycled by filtering through 0.45 and 0.1 μm integrated Nucleopore filters. A mix of fluorescent beads (Polysciences microspheres of 1.00 ± 0.03 μm, 1.6 ± 0.1 μm, 2.9 ± 0.1 μm, and 10.3 ± 0.4 μm) was regularly analyzed to ensure quality control, reliable analyses and calibrate cell sizes. Cells sharing similar optical fingerprints were manually grouped in clusters on a set of 2D projections (cytograms) in the Cytoclus software (Cytobuoy b.v.).

### Estimation of cell volume

**Figure 2:**
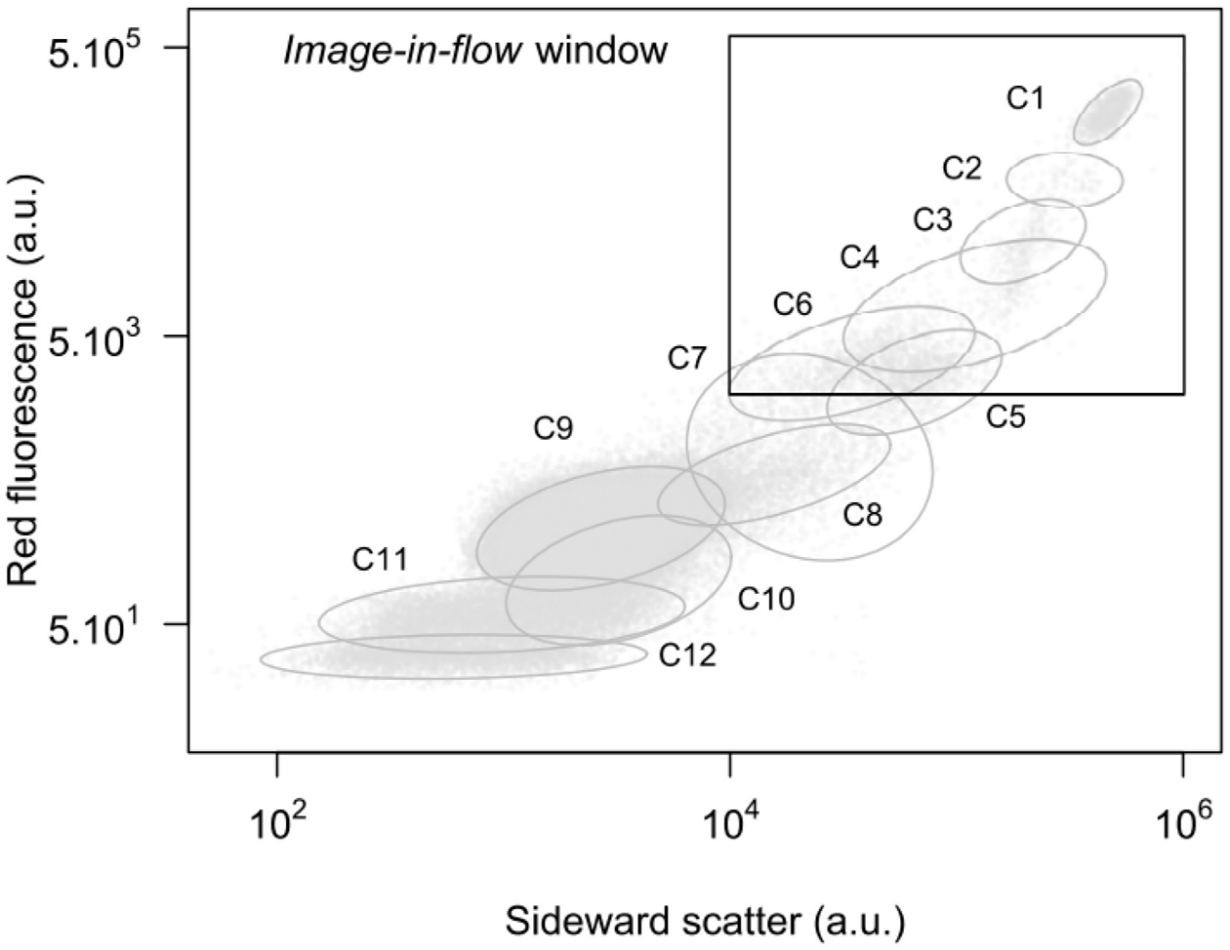
**Cytogram total red fluorescence vs. total sideward scatter (a.u.) with 95% confidence ellipses for the various clusters optically resolved during this study**. The “image-in-flow” window used to trigger the picture acquisition is represented by the black square.

**Figure 3:**
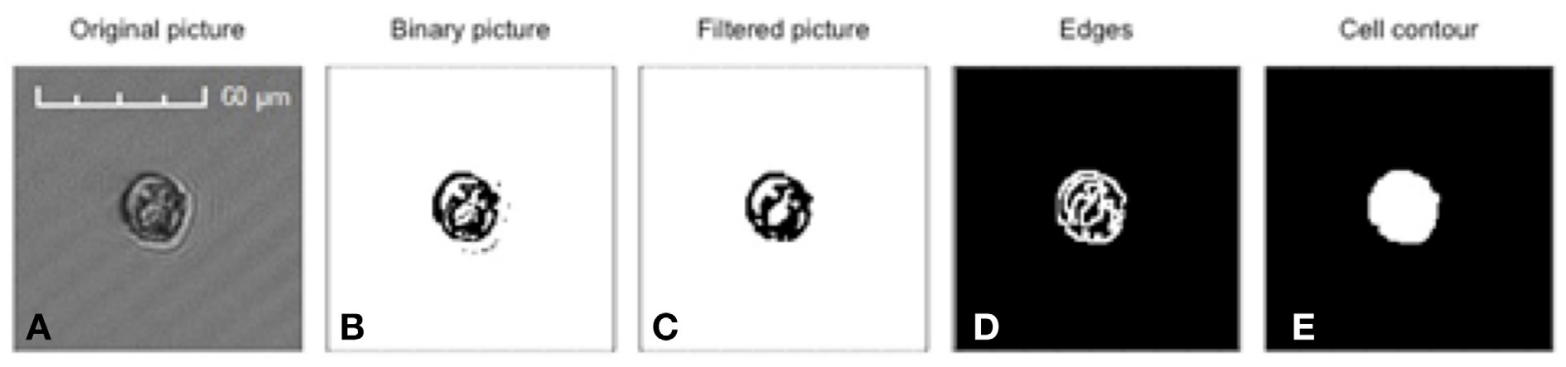
**Image processing of a Gymnodinium picture performed by the Fiji software in order to extract the cell dimensions. (A)** Original picture **(B)** Binary transformed picture **(C)** Filtered binary picture **(D)** Edges detection between pixels **(E)** Contour selection of the cell.

**Figure 4:**
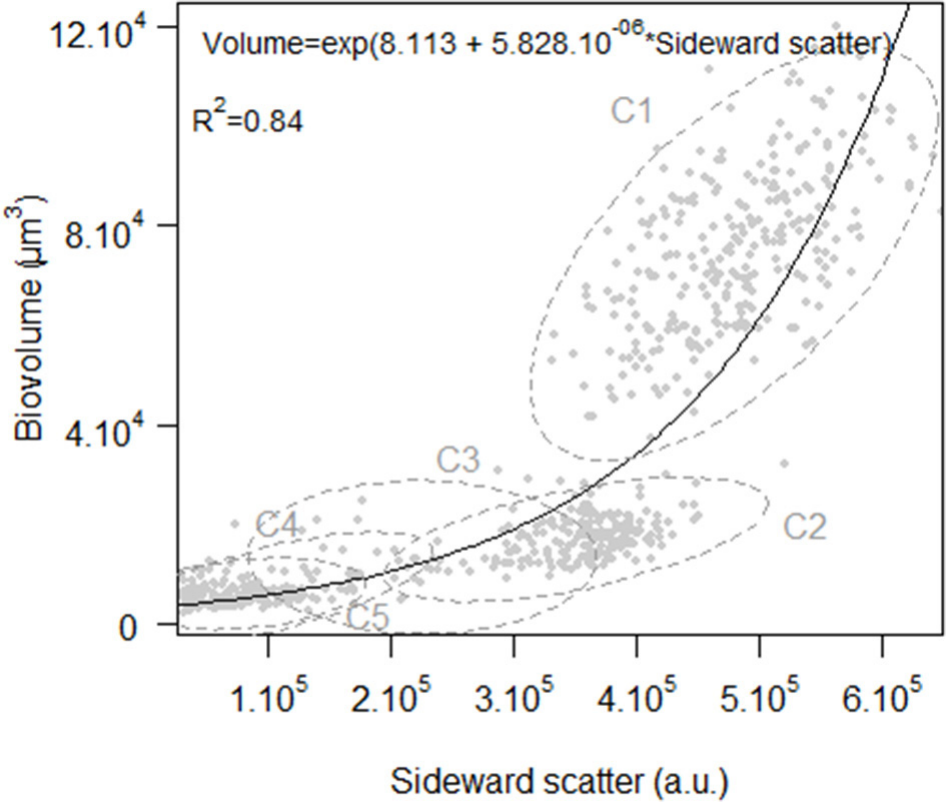
**Exponential relationship between total sideward scatter and biovolume of the cells pictured by the “image-in-flow” camera (*n* = 693) mounted on the Cytosense**.

The Cytosense used in this study is equipped with an “image-in-flow” device controlled by the CytoUSB software (Cytobuoy b.v.). During this October 2011 survey, cells displaying the highest red fluorescence and scatter intensities (a.u.) were manually gated within a targeted window where a maximum of 60 pictures per analysis (current limitation of the software) were taken (Figure 2). These pictures were used to identify and estimate the dimensions of the cells in the different clusters. Area, length, and width were automatically calculated after image processing of given files containing the original pictures (Figure 3A) using the Fiji software, an open source version of the ImageJ software (Schindelin et al., 2012). The first step of the image processing was to automatically segment the picture into foreground (cells) with pixels values of 255 (black) and background with pixels of 0 (white) (Figure 3B). Noise was filtered (Figure 3C) and edges between pixels' value were selected as illustrated by Figure 3D. Finally we used the external edge as the contour of the cell (Figure 3E) to compute the measurements. Biovolume of all the pictured cells was estimated considering either spherical or ellipsoidal model according to cell shapes. An exponential function explained 84% of the variance between cell volume and SWS (Figure 4).

### Estimation of *in situ* growth rates

To estimate phytoplankton *in situ* growth rates, we used the size-structured population model described in Sosik et al. (2003) by applying the regression between cells' volume and sideward scatter intensities to all the cells within a specific cluster. In this model, cells are classified into several size classes according to their dimensions at time *t*. The number of classes, *m*, was chosen in order to cover the entire observed biovolume spectrum from *v_min_* to *v_max_*. Classes were logarithmically spaced as follows:

For i=1,2,…,m vi = vmin·2(i − 1)Δv

with Δ*v* constant.

At any time *t*, the number of cells in a size ranging from *v_i_* to *v*_*i* + 1_, noted *N_i_*(*t*), was projected to *t* + *dt* via matrix multiplication so that:

N (t + dt) = A (t) ·N(t)

The elements *a_ij_*(*t*) of the matrix ***A*** correspond to the fraction of cells of class j at time *t* that becomes cells of the class i at time *t* + *dt*. Changes in cell size are linked to two phases of the cell cycle: the size growth during the interphase and the size decrease after the mitosis. In *dt*, one cell can either go to the direct next size class, that is to say from *v_i_* to *v*_*i* + 1_ through cellular growth, or can become a cell of half its original size after division. If in *dt* one cell neither grows nor divides, it remains in the same size class (i.e., stasis).

Adjustments have been made in order to apply the model described in Sosik et al. (2003) for growth rate estimations from the dynamics of the clusters in this study. Basic hypothesis were adapted to find the best fit with natural data. Elements of the -1 shifted main diagonal, corresponding to the fraction of cells that grew between *t* and *t* + *dt* and reached the next size class were directly calculated from:

$$a_{i\;+\;1,i}\left(t\right)=\gamma \left(t\right)$$

However, elements of the Δ*v* shifted main diagonal, corresponding to the fraction of cells that divided between *t* and *t* + *dt* and reached the size class of half their size, were counted from the fraction of cells that were not currently growing:

$$a_{i,i\;+\;1/\Delta v}\left(t\right)\;=\;2\cdot \delta_{i\;+\;1/\Delta v}\cdot \left(1-\gamma \left(t\right)\right)$$

Elements of the main diagonal remained those fractions of cells that has neither grown nor divided between *t* and *t* + *dt*:

$$a_{i,i}\left(t\right)=\left(1-\delta_{i}\right)\cdot \left(1-\gamma \left(t\right)\right)$$

The probability of cells growing to the next size class was given by an asymptotic function, assuming that it only depends on the light intensity necessary to photosynthesis:

$$\gamma (t)=\gamma_{max}\cdot \left(1-e^{-E\left(t\right)/E^{*}}\right)$$

with *E*, the light intensity, γ_*max*_ and E^*^ diel constants.

The probability of cells dividing was given by a log-normal density function, assuming that it depends on the size spectrum of the vegetative cells only:

δi=a·e−(logvi − logv¯)2/2·logσ2

with *a*, *v* and σ diel constants.

This model was chosen to ensure that only the size distribution of vegetative cells will account for the estimation of the population growth rates. Even if dinoflagellates species are haplontics, the coexistence of asexual and sexual forms is well documented and expected after sediment resuspension induced by turbulent mixing (Kremp and Heiskanen, 1999). Since growth rates are manifestations of the asexual life cycle, the incorrect use of sexual stages could inaccurately estimate division rates. However, dinoflagellate stages are generally heteromorphic (Pfiester and Anderson, 1987). Vegetative and planozygote/cyst cells can display distinct size (Von Stosch, 1973; Walker, 1982; Anderson et al., 1983; Blackburn et al., 1989; Figueroa et al., 2008) so the Gaussian function should enable either to restrain the size spectrum if sexual or alternative (G0) forms are present or to take into account the entire spectrum if not.

*In fine*, growth rates can be calculated according to the following formula:

$$\mu =\ln\left(\frac{\overset{m}{\underset{i\;=\;1}{\sum }}{\hat{N}}_{i}\left(24\right)}{\overset{m}{\underset{i\;=\;1}{\sum }}N_{i}\left(0\right)}\right)$$

with *N(0)* the initial observed size distribution, at *t* = 00:00 and $\hat{N}$ (24) the fitted size distribution at *t* = 24:00.

The parameters of the projection matrix ***A*** were those that minimize the sum of squared differences between observed and projected proportion distributions, ***w***, ever 24 h. Either the entire distribution or only the median of the distribution was used to optimize the function:

$$\sum_{t\;=\;1}^{24}\sum_{i\;=\;1}^{m}{\left(w\left(t\right)-\hat{w}\left(t\right)\right)}^{2}$$

### Statistical analysis

Statistical analysis and modeling codes were performed using the R freeware (http://www.r-project.org). The time series of mean wind speed have been fitted using a linear regression (l m function, CRAN) in order to select the window within which the temporal trend explained most of the variation of wind speed. Significance of trends of hydrological variables in this window have been estimated by comparing linear mixed-effects regression models (lmer function, lme4 library, CRAN) adapted to pseudoreplication of observations in time series (Pinheiro and Bates, 2000). To account for the strong serial correlation between measurements, random effects have been modeled as deviations of intercepts between factors grouping measurements equivalent to one period in each time series. Periodicity has been studied using discrete Fourier transforms (fft function, CRAN) on detrended time series. The maximum amplitude associated with the harmonic frequency equivalent to one period determined by Fourier analysis have been applied to differences between observed variables and non-linear trends fitted by local polynomial regressions (loess function, CRAN). We compared, with a likelihood ratio test (anova function, CRAN), models estimated by maximum likelihood with fixed effects formulated as seasonal cycles with and without a linear time trend:

$$\begin{array}{l} \text{Y}(t)=a_{0}+a_{1}\text{index}(t)+a_{2}\sin(2t\pi )+a_{3}\cos(2t\pi )+\varepsilon (t)\text{ and }\\ \text{Y}(t)=a_{4}+a_{5}\sin(2t\pi )+a_{6}\cos(2t\pi )+\varepsilon (t)\; \end{array}$$

with *(t)* scaled to one period, and ε(*t*) residuals iid~ N(0,σ^2^). For these models, residuals did not reveal any deviations from homoscedasticity, normality, and temporal dependency as tested by the autocorrelation function (acf function, CRAN). Correlation coefficients between environmental variables and cluster abundances were represented with a PCA (FactoMineR library, CRAN). Cross-correlation between wind speed time series and all the clusters abundance time series were performed to estimate the lag time between respective maxima. Negative lag time correspond to cluster abundance maximum prior to the wind event and inversely for positive lag time.

## Results

### Optical resolution of the flow cytometry clusters

Up to 12 flow cytometry clusters of phytoplanktonic cells (arbitrary labeled C1–C12) have been resolved over the sampling period. Each cell contributed to a particular cluster on the basis of the distinct optical properties recorded. Clusters showed a proportional relationship between SWS (correlated to cell biovolume, 0.82, *p* < 0.001) and red fluorescence emitted by chlorophyll pigments. Almost all the clusters were well discriminated on red fluorescence vs. sideward scatter cytogram (Figure 2). With the same level of red fluorescence, clusters C5 and C8 emitted more orange fluorescence than clusters C6 and C9, respectively. The size spectrum of the cells ranged within the pico-, nano-, and microphytoplankton size classes. Smallest cells were observed in the cluster C12 (mean 0.9 ± 0.1 μm) and the biggest cells belonged to cluster C1 (mean 56.4 ± 12.2 μm). Clusters C1–C4 were composed of different dinoflagellates species identified as *Akashiwo sanguinea* (Hirasaka), *Prorocentrum micans* (Erhenberg), *Scrippsiella* sp. (Balech) and *Gymnodinium* sp. (Stein), respectively over the all sampling period. Clusters C5–C10 were assigned to nanophytoplankton with no visual identification possible by the “image-in-flow” pictures due to the lack of resolution of the pictures. Picophytoplankton was represented by clusters C11 and C12 with mean length estimated by the calibrated beads at 1.1 ± 0.1 μm and 0.9 ± 0.1 μm.

### Phytoplanktonic community dynamics

#### Environmental variables

**Figure 5:**
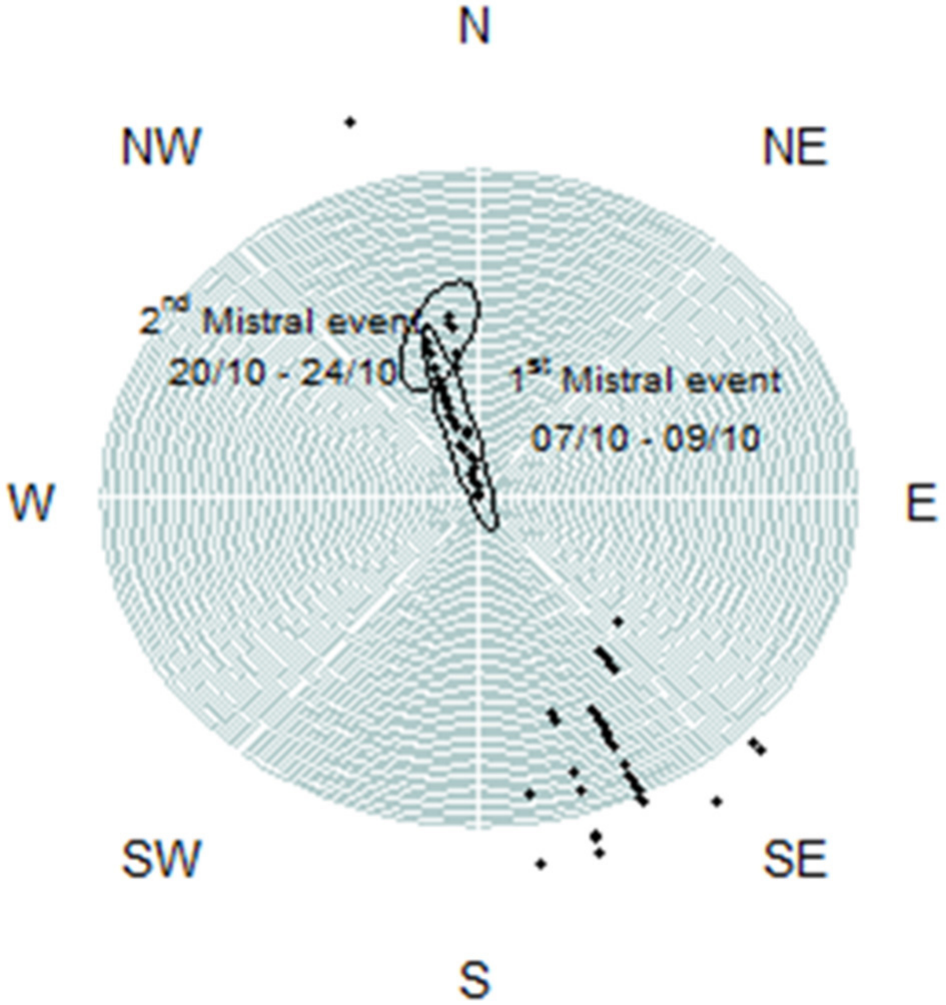
**Distribution of wind direction during October 2011 with gusts exceeding 20 m/s**.

**Figure 6:**
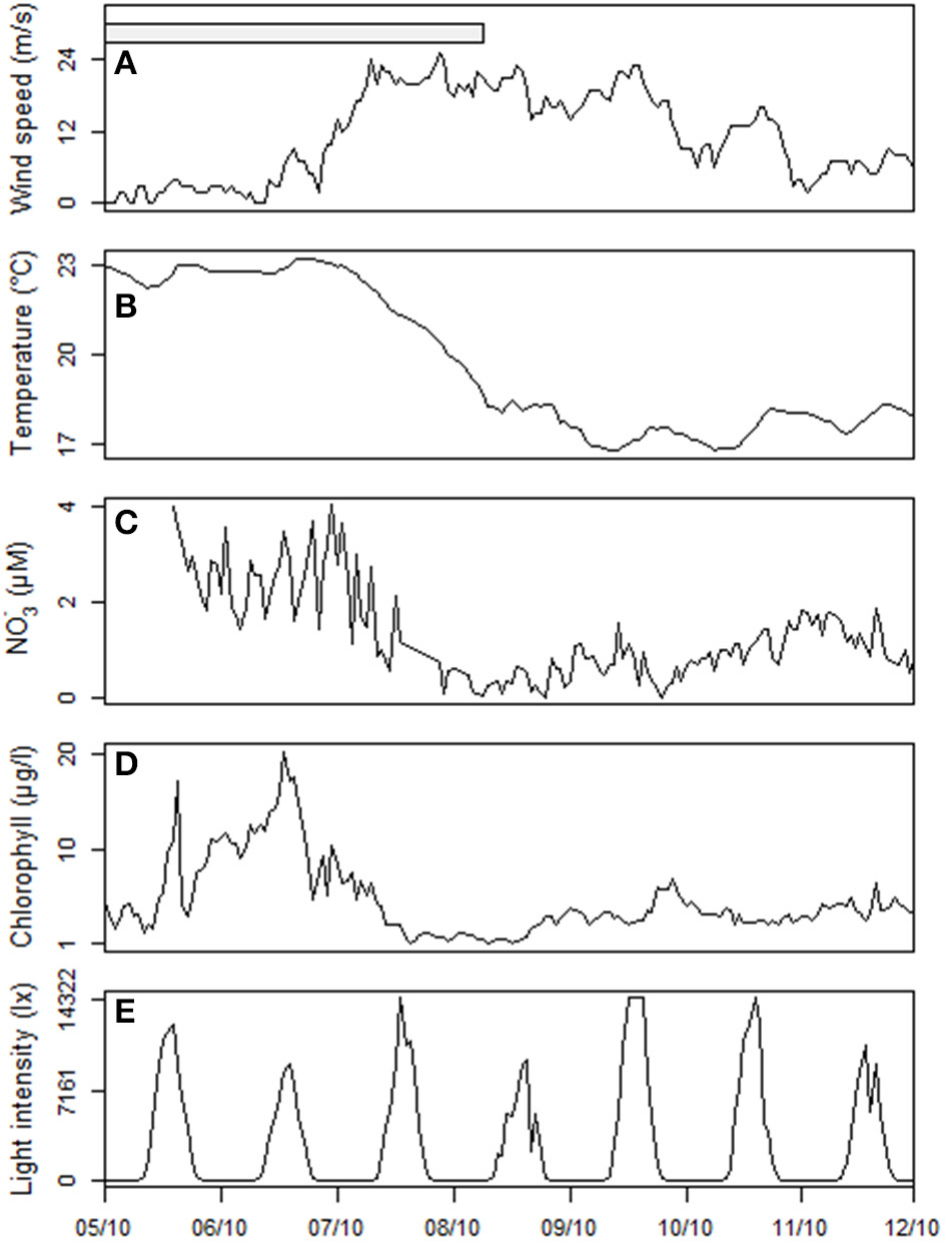
**Dynamics of the hydrological variables before, during and after the strong wind (Mistral) event. (A)** Wind speed, **(B)** Temperature, **(C)** *NO*^−^_3_ concentration, **(D)** Chlorophyll concentration, and **(E)** Light intensity.

**Figure 7:**
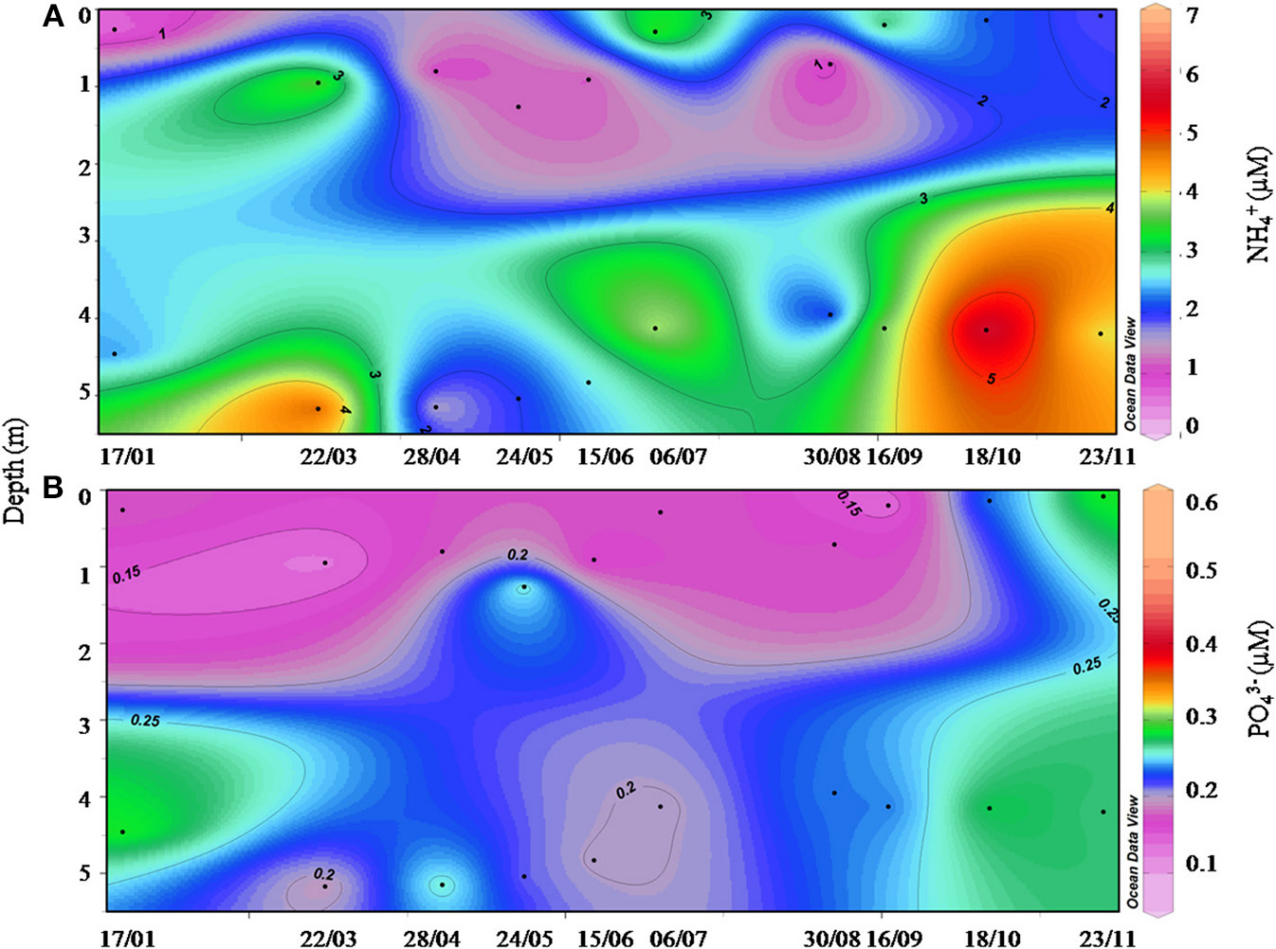
**Vertical distribution of (A) ammonium and (B) phosphates concentrations measured by the GIPREB at surface and bottom layers at the hydrological station in the Vaïne lagoon in 2011**.

During October 2011, the wind was the main forcing on the hydrological state of the Berre lagoon and more particularly the intense Mistral blowing from the North (330–360°), with mean speeds exceeding 20 m/s (Figure 5). Turbulent mixing of the water column increased as gusts reached 18.7 ± 3.2 m/s between October 7th and 9th (Figure 6A). During these 2 days, all the hydrological variables showed significant trends with the exception of *in situ* light intensity [χ^2^_(1)_, *n* = 79, *p* < 0.05]. Mean water temperature measured at the sampling point cooled by 5.2°C between October 05th and 11th (Figure 6B). Salinity distribution showed a wider range of variation during the Mistral event, suggesting a gradient between surface and bottom layers prior to the wind: difference between 0.05 and 0.95 percentiles was 0.19 on October 6th and 0.59 on October 8th. On September 16th and October 18th, the nutrient vertical profiles measured by the GIPREB at surface and bottom depths displayed higher concentrations of recycled nutrients (*NH*^+^_4_, *PO*^3−^_4_) at the bottom of the lagoon, suggesting the stratification of the water column prior to and after the first Mistral event (Figure 7). Variation in nitrate concentrations was similar to temperature (Figure 6C) with a negative trend of –0.04 μM per hour [χ^2^_(1)_ = 17.2, *n* = 79, *p* < 0.001] which led to a minimum of 0.29 μM on October 9th at 19:00. Chlorophyll concentration reached 20.96 μg/l on October 6th 13:00 (Figure 6D). After the maximal gust of 25 m/s, the mean value dropped to 3.97 μg/l. Despite the decrease in chlorophyll concentration the *in situ* light intensity was not significantly different before or during the Mistral event [χ^2^_(1)_ = 0.07, *n* = 79, *p* = 0.8]. The photoperiod was between 08:00 and 19:00 and the light intensity was always maximal at 12:00 (Figure 6E).

#### Community response to the wind event

**Figure 8:**
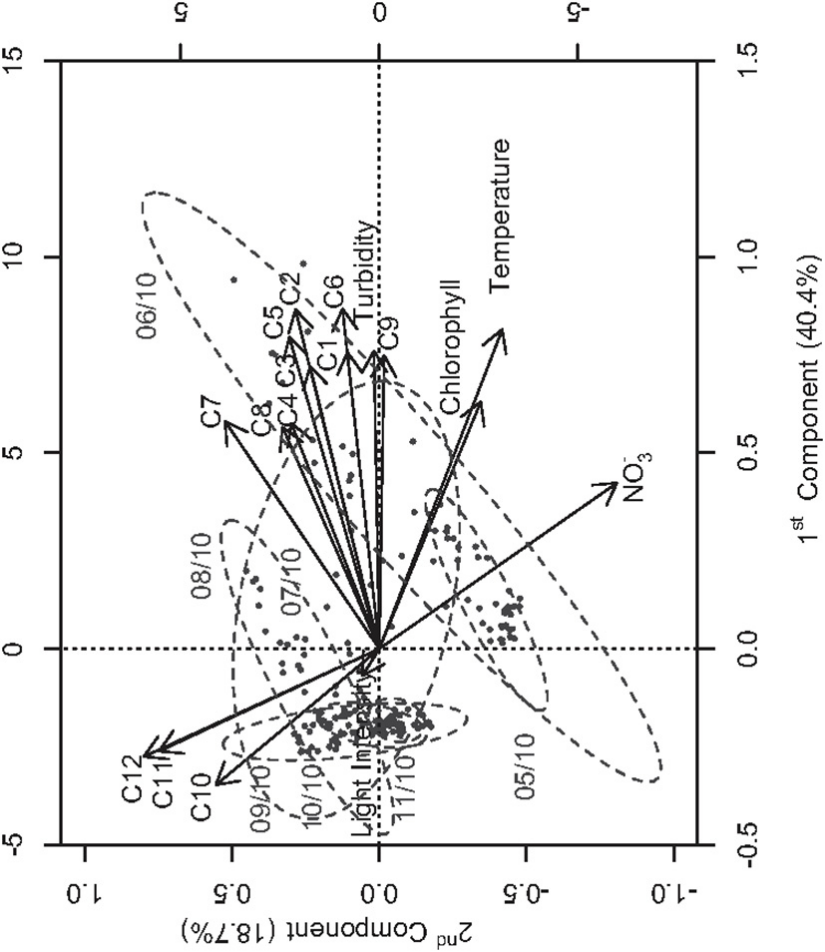
**Principal component analysis performed on the dataset**. Projection of the observations (gray points, i.e., dates from 10/05/11 00:00 to 10/11/11 23:00) and the variables (arrows, i.e., hydrological variables and clusters abundance) on the two first principal components of the PCA (59.1 % of the total variance).

**Figure 9:**
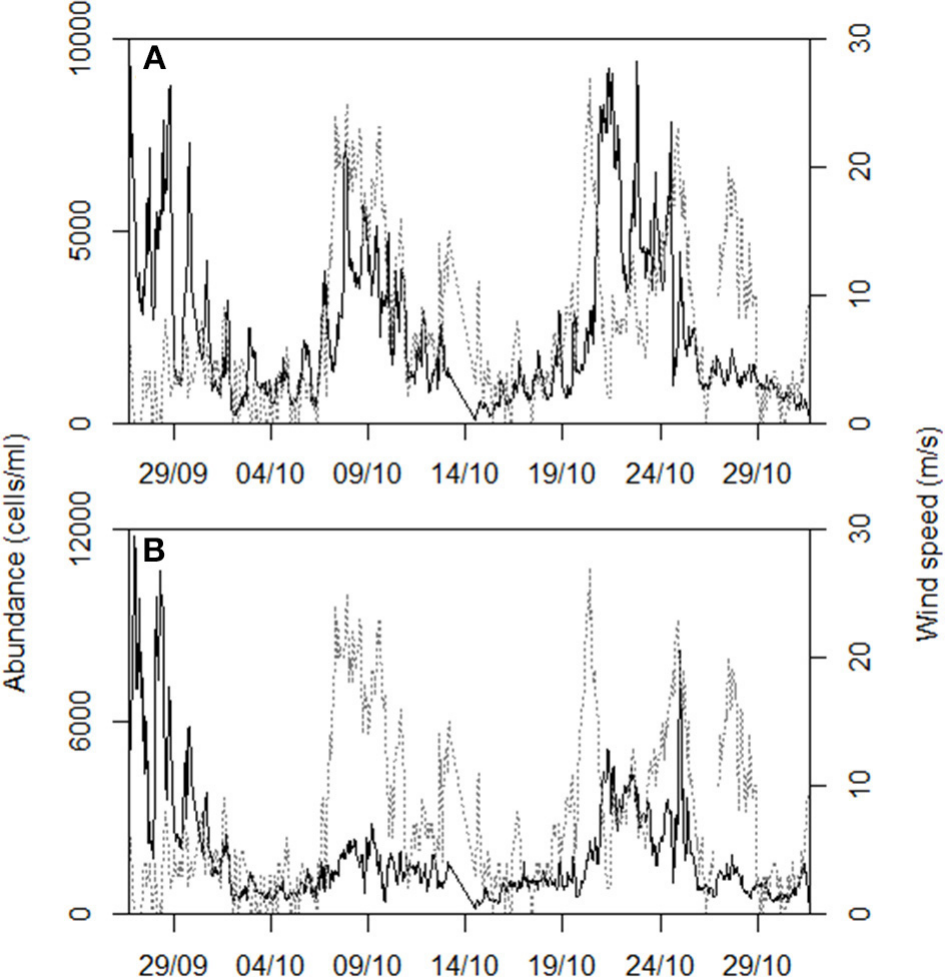
**Time series of the (A) C11 cluster and (B) C12 cluster abundances (black line) during the sampling period, characterized by two strong Mistral event (gray dotted line)**.

A Principal Component Analysis was performed to analyze the multivariate time series of hydrological variables and cluster abundances. The main relationships between phytoplankton concentrations and the environment were well represented on the two first components, which explained 59.1% of the total variance. High chlorophyll concentration was observed simultaneously with the occurrence of higher abundances of clusters C1–C9 (Figure 8). The increase of chlorophyll concentration and turbidity in the Berre lagoon was phased with the sudden proliferation of clusters C1–C3, C5, and C8 (correlation coefficient 0.36, 0.46, 0.31, 0.50, and 0.51, respectively, *p* < 0.001), as well as the presence of C9 cells. C9 was the most abundant cluster with mean concentration 17,024 ± 10,880 cells/ml between October 05th and 06th and a maximum of 51,142 cells/ml on October 05th 20:00. All of these clusters were more abundant before the intensification of the Mistral wind speed (Table 1). The lag time between maximum wind speed and abundances mainly ranged between 24 and 48 h. Turbidity was correlated to high temperature (0.63, *p* < 0.001) and high nitrate concentration (0.26, *p* < 0.001). Conversely, picophytoplankton cluster (C11 and C12) concentrations increased less than 1 h after the wind speed rose (correlation coefficient 0.72 and 0.60, *p* < 0.001). Coefficients of cross correlation were thus maximal for the 0 h lag time between wind speed and abundance time series. Mean abundance was 1482 ± 1009 cells/ml for C11 and 923 ± 317 cells/ml for C12 between October 05th and 06th and reached 3787 ± 1557 cells/ml and 1690 ± 411 cells/ml during October 07th and 08th. The increase in these cluster concentrations was reproduced during a second strong Mistral event (Figure 9), with an incremented amplitude. The abundance of the *Gymnodinium* cluster did not significantly change between October 05th and 11th. The 3rd and 4th principal component that explained 8.7% and 6.7% of the total variance respectively (data not shown) showed the negative correlation between light intensity and turbidity (–0.14, *p* < 0.1) and the positive influence of light on chlorophyll concentration mostly before the Mistral intensification (0.36, *p* < 0.1).

**Table 1:** **Maximum cross correlation coefficient and associated lag time of wind speed and cluster abundance time series between October 5th 00:00 and October 11th 23:00**.

**Clusters**	**C1**	**C2**	**C3**	**C4**	**C5**	**C6**	**C7**	**C8**	**C9**	**C10**	**C11**	**C12**
Coefficient	0.57	0.62	0.62	0.48	0.62	0.60	0.63	0.73	0.57	0.46	0.96	0.92
Lag (h)	−32	−30	−28	132	−30	−39	−14	−26	−48	49	0	0

### Dynamics of the phytoplanktonic populations'

#### Gymnodinium dynamics

**Figure 10:**
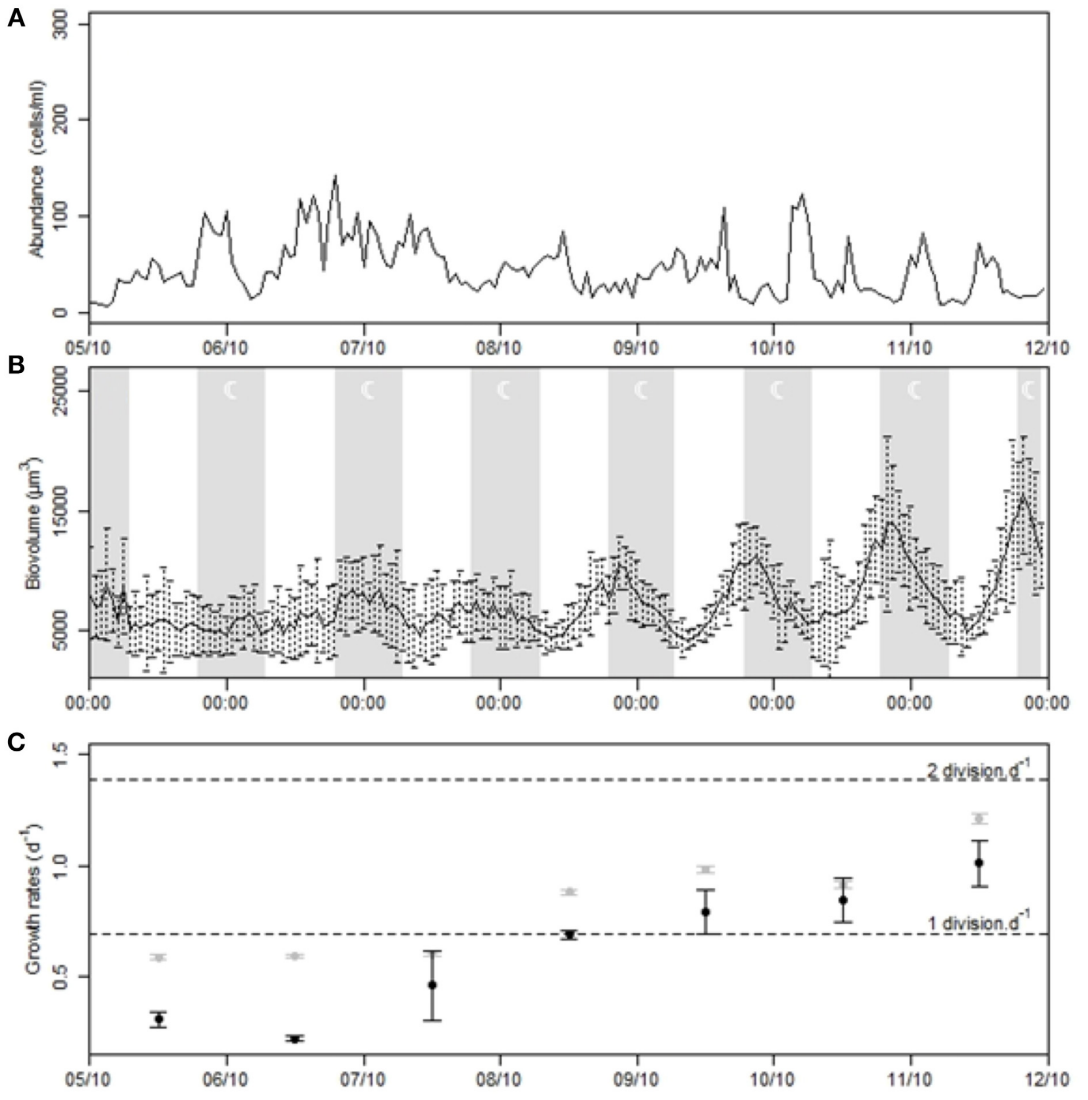
**Time series of the *Gymnodinium* cluster during the strong Mistral event. (A)** Population dynamics. **(B)** Variation of the mean cells biovolume. **(C)** Estimations of the *in situ* growth rate provided by the ratio between maximal and minimal mean diel biovolume (gray points) and the model (black points). Error intervals correspond to growth rate estimates after applying the biovolume regression model with standard errors.

**Figure 11:**
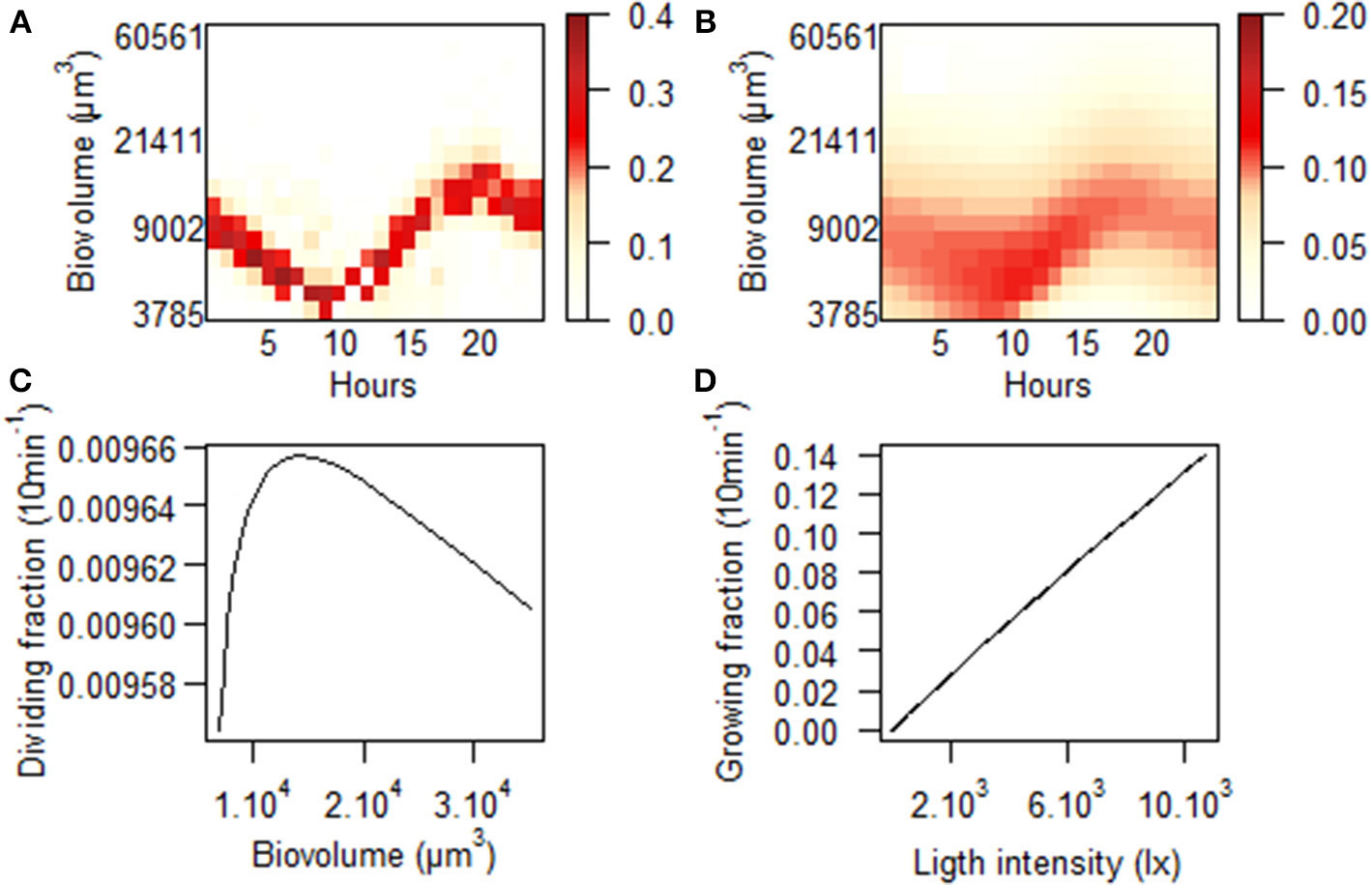
**Output of the size structured population model applied to cluster C4 on October 11th 2011: Diel variations of the (A) observed and (B) theoretical distributions of cell proportions in each size class**. The theoretical distribution was estimated only on the basis of two cell cycle processes, division and size growth with probability depending on **(C)** the biovolume of the cells and on **(D)** the incident light intensity respectively.

From the pictures taken by the “image-in-flow” device, cluster C4 has been identified as *Gymnodinium* sp. Its dynamics was quasi-periodic during the Mistral event (Figure 10A). The concentration was marked by several peaks of abundance with a mean of 49.9 ± 28.8 cells/ml. The highest concentration reached 142.4 cells/ml on October 6th at 19:00 and the abundance regularly dropped down below 50 cells/ml. Between October 05th and 09th, a high abundance was generally observed at the end of the day, but after the lull in the wind two distinct peaks were observed each day. On October 10th, maximal concentrations (of 122.8 and 79.2 cells/ml) were measured at 05:00 and 13:00, respectively. On the 11th, they were measured at 03:00 and 13:00, with 82.8 and 71.6 cells/ml, respectively. The same day, the maximum mean biovolume of the cells was 3 times greater than the minimum, passing from 4852–16,294 μm^3^ (Figure 10B). During this period, cells were highly phased within cluster C4, making the estimation of growth rates based on the biovolume distribution more reliable (Figure 11). To apply the model and compare the observed (Figure 11A) and theoretical (Figure 11B) distribution of the biovolume, *v_min_* and *v_max_* were set to 3785 μm^3^ and 59,874 μm^3^, dt to 10 min, Δ*v* to 1/4 and *m* to 17. The mean cell biovolume increased 1 h after dawn until approximately 20:00 (Figure 11D) before it progressively decreased due to mitotic divisions (Figure 11C). The amplitude of these two processes varied before, during and after the wind event so that growth rates ranged between 0.22 d^−1^ (<1 division per day) and 0.85 d^−1^ (> 1 division per day) (Figure 10C).

#### Dynamics of cluster C5

**Figure 12:**
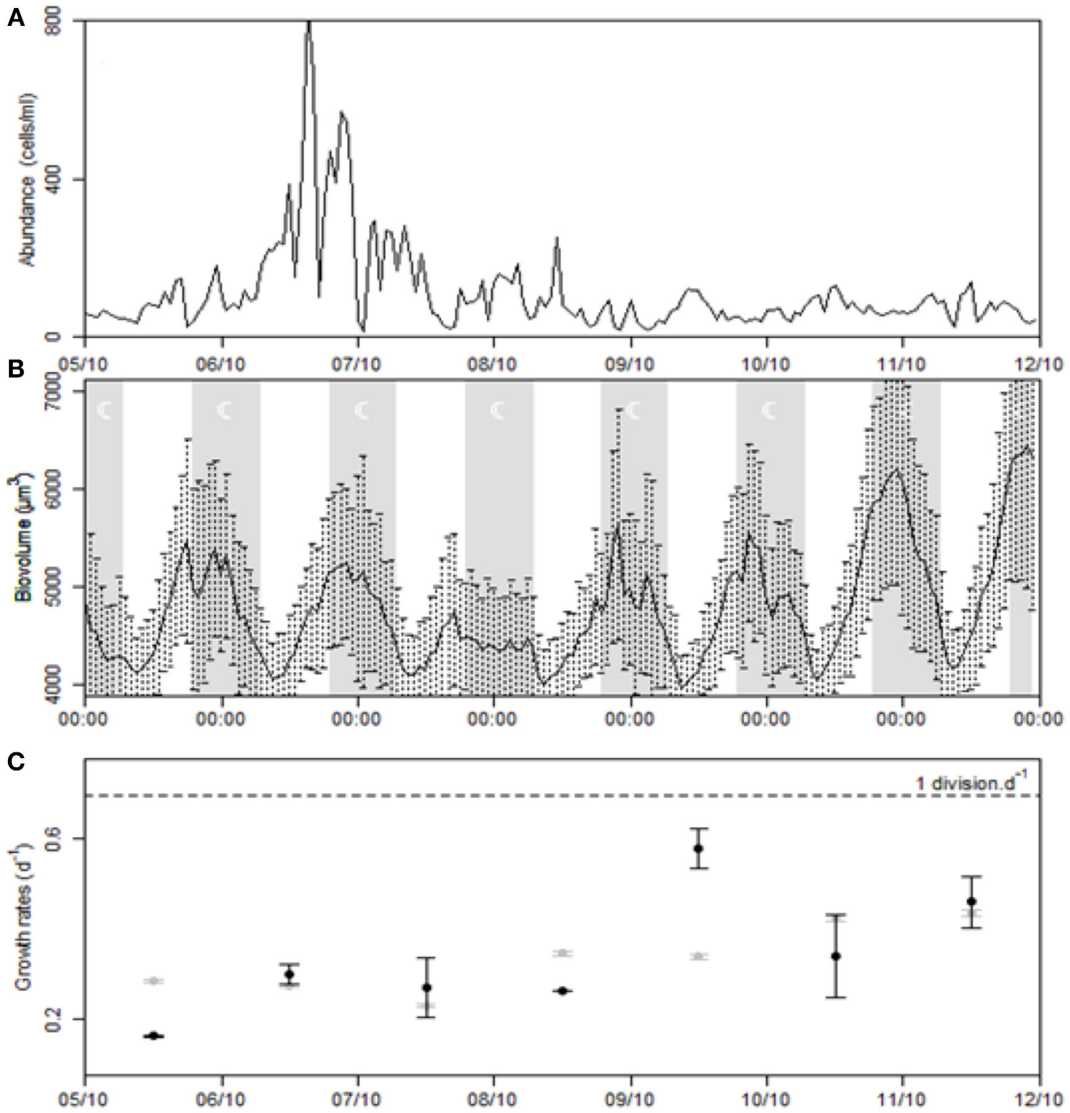
**Time series of the cluster C5 during the strong Mistral event. (A)** Population dynamics. **(B)** Variation of the mean cells biovolume. **(C)** Estimations of the *in situ* growth rate provided by the ratio between maximal and minimal mean diel biovolume (gray points) and the model (black points). Error intervals correspond to growth rate estimates after applying the biovolume regression model with standard errors.

Cells forming the cluster C5 could not be identified on the pictures. It was one of the clusters that showed a sudden peak of abundance on October 6th, reaching up to 838 cells/ml at 15:00 (Figure 12A). Two hours later, the concentration was 101 cells/ml and remained at a mean value of 85.9 ± 57.7 cells/ml. The cells were well phased with the light/dark period between October 5th and 11th as indicated by the mean cell biovolume that exhibited a clear diel variation in relation to the cell cycle (Figure 12B). Cell size began to increase 1 h after dawn (09:00), until a few hours after dusk (20:00–23:00). The ratio of max/min mean biovolume ranged from 1.26 on October 7th to 1.55 on October 11th. Between these dates, growth rates progressively increased with regard to the variation of mean biovolume amplitude and reached 0.46 d^−1^ on October 11th (Figure 12C). To apply the model to cluster C5 and compare the observed and theoretical distribution of the biovolume, *v_min_* and *v_max_* were set to 3707.6 μm^3^ and 16,017.6 μm^3^, *dt* to 10 min, Δ*v* to 1/9 and *m* to 20.

## Discussion

### Phytoplankton in the berre lagoon

Because of high nutrients inputs and regeneration (Gouze et al., 2008), the primary production in the Berre lagoon has always been high compared to others Mediterranean lagoons (Minas, 1976; Kim, 1983). High production rates leading to blooms combined with great diversity have made this site a unique area for phytoplankton study.

In October, these blooms have been observed due to persistent summer-like temperatures, causing a thermal stratification of the water column, and the resurgence of nutrients supply by the power plant activities. Before the freshwater discharges were regulated by the power plant, algal proliferation was almost monospecifically dominated by red tide dinoflagellates such as *Prorocentrum minimum*. However, the wind regimes occurring in this region play a major role in mixing and oxygenating the water column. This limits the negative impact of hypoxia that could be induced by the heterotrophic activity. The Mistral is a strong Northerly wind that generally drives the superficial layers of the Berre lagoon toward the Caronte channel to join the Mediterranean Sea (Ulses et al., 2005). It causes the homogenization of the water mass, mixing warm surface layer and colder bottom layer (Nerini et al., 2001). All these forcings drove the phytoplanktonic community to evolve in order to develop within the range of temperature and salinity gradients induced by wind, rivers and freshwater discharges. However, since the regulation of discharge imposed on the hydroelectric plant, species richness has increased (Raimbault et al., 2013). Diatoms (*Chaetoceros* sp., *Pseudo-Nitzschia* sp., and *Thalassionema* sp.), dino- and nanoflagellates have been sampled at 10 hydrological stations and identified by microscopy and flow cytometry (data not shown), technique recently implemented in the monitoring strategy of the GIPREB. Flow cytometric analyses by an autonomous *in situ* flow cytometer have revealed more phytoplankton clusters in the lagoon than in the Bay of Marseille, in the NW Mediterranean Sea (Thyssen et al., 2011). In 2005–2006, 7 clusters were resolved in the Bay of Marseille assigned to pico (2 clusters) and nanophytoplankton (5 clusters) compared to 12 clusters identified in 2011 in the Berre lagoon at the sampling point. Two clusters were assigned to picophytoplankton, 6 clusters of nanophytoplankton and 4 of microphytoplankton. In the microphytoplanktonic clusters, cells were identified as red tide dinoflagellates species such as *Akashiwo sanguinea*, *Prorocentrum micans*, *Gymnodinium* sp., and *Scrippsiella* sp. The first two are eurythermale and euryhaline species, able to grow within temperature (10–30°C) and salinity (10–40) gradients (Matsubara et al., 2007; Dhib et al., 2013).

### Community and production changes to a strong mistral event in the berre lagoon

Offsets of phytoplanktonic production are calculated from the loss and growth rates of natural populations. Loss in net productivity at a fixed point can result from trophic interactions such as grazing and viral lysis as well as physical transport (Riley et al., 1949). For our experiment, the sampling strategy for high frequency characterization of the phytoplankton community has been limited to one depth at a fixed station, and thus represents only a partial view of the response of phytoplankton clusters in the lagoon. However, several 3D hydrodynamical models simulate the circulation in the station location as confined within the Vaïne lagoon in case of a strong Mistral event (Leredde et al., 2002; Alekseenko et al., 2013). Moderate turbulent mixing is usually beneficial for large non-motile cells of phytoplankton because it counterbalances their sedimentation and prevents their exportation toward the aphotic layer (Margalef, 1978). The classical succession scheme induced by temporal or spatial ergoclines (Legendre and Demers, 1985) predicts the outbreak of diatoms followed by dinoflagellates when nutrients are depleted in the surface layers. Because of both inputs of freshwater and seawater, maintaining the haline gradient between surface and bottom layers, the deepest part of the Berre lagoon is almost permanently stratified (Nerini et al., 2001). Only strong gusts of wind (>10 m/s) allow the homonogeneization of the water column. In October 2011, automated flow cytometry analyses prior the strong Mistral event revealed the sudden proliferation of red tide dinoflagellates as well as nanophytoplankton clusters. Dinoflagellate species regroup complex microorganisms. Their life cycle implying sexual, asexual, and resting stages as well as their trophic strategy can both justify the potential of dinoflagellates to outcompete other phytoplanktonic species (Schnepf and Elbrachter, 1992; Kremp, 2013). When inorganic compounds are not sufficient in their environment, dinoflagellates can swim in order to have access to the deeper nutrient pool or ingest small prey in food vacuoles if they are mixotrophs (Stoecker, 1999). The proliferation of dinoflagellates during our experiment thus supports the idea that they are K strategists relying on the microbial loop for the uptake of regenerated nutrients (Smayda, 1997; Pitcher et al., 1998; Kudela et al., 2008). On the contrary, high frequency sampling revealed that picophytoplankton clusters might show a reactive capacity superior to other groups such as diatoms, and so be defined as pioneer species. Autonomous sampling has already indicated that picophytoplankton could be the first to develop following a strong wind event (Thyssen et al., 2008). Higher assimilation rates give these small cells an advantage compared to larger species accounting for new production as well (Eppley et al., 1969; Aknes and Egge, 1991). In theory, the concentration of such r strategists should increase within a short lag time after the perturbation. In this study, clusters C11 and C12 started to multiply less than 1 h after the wind speed increase. This behavior was reproduced during a second strong Mistral event, which also favored their proliferation soon after the weather lull, but likely prevented the reappearance of the following successors, accounting for regenerated production fuelled by the organic matter recycling (Legendre and LeFèvre, 1989).

### Growth rate estimations

As sideward scatter is strongly correlated to cell size (Ackleson and Spinrad, 1988; Simon et al., 1994), determination of cell optical properties can provide information about the life cycle of micro-organisms (Kiefer et al., 1979). The model developed by Sosik et al. is based on the full size distribution that varies during the sequential phases of the cell cycle. Therefore, cells will grow with the support of photosynthesis and divide according to their size. In an attempt to accommodate these simple assumptions to clusters observed in this study, we tested an alternative model for division, relaxing the hypothesis of the strict increase of division rates with biovolume. However, caution should be obviously taken with *in situ* growth rates estimations of dinoflagellate populations, particularly with regard to life stages differentiations (Cetta and Anderson, 1990; Gisselson et al., 1999). No inhibitory effect of high light intensity or UV exposure was expressed in the growth model since the daily red fluorescence variation did not exhibit any depression during the day (data not shown), as can be observed when quenching occurs (Jacquet et al., 1998). This model fitted C4 and C5 clusters size distribution patterns that were, as for dinoflagellates (Sweeney, 1959), phased to the photocycle. Cellular growth began ~1 h after dawn and continued briefly after dawn, whereas division mainly occurred during the night, in agreement with the literature (Weiler and Chisholm, 1976). As a greater proportion of cells was in the G1 phase, we changed the dependence relation between division and growth to reflect that division mainly occurred when cellular growth was no more dominant. After the Mistral event, cells reached higher dimensions during the photoperiod, suggesting a possible light limitation. Since duration of the interphase and mitotic divisions was unchanged, growth rates naturally increased. Cells in the *Gymnodinium* cluster underwent from less than one division to more than one division per day after October 9th. The maximal growth rate was at 0.85 d^−1^ on October 10th. Growth rates of the C5 cluster were always inferior to 0.69 d^−1^ but they progressively rose to 0.57 d^−1^.

Clusters C1–C3 were not abundant enough relatively to the volume analyzed to ensure a reliable application of the model. The size variation did not show any clear pattern phased to the photoperiod. The shape of size histograms was constant during the day, giving evidence of asynchronous populations (Campbell and Yentsch, 1989). Asynchrony of dinoflagellates appears when spatial migration is triggered by physiological needs, local current, cyst bed or wind rather than by the light/dark cycle (Ralston et al., 2007). Nutritional migration strategy over photo/geotaxis in natural populations leads to deep chlorophyll maximums which coincide with the primary production, near the nutricline (Cullen, 1982). However, synergy between nutrient availability and irradiance needs depends not only on hydrodynamics but also on internal cells' nutritional status, reinforcing the variability within populations (Ji and Franks, 2007). In some particular ecosystems, the vertical distribution of dinoflagellates is typically bimodal, with a second abundance peak at the surface (Townsend et al., 2001). Even if diel size variation can be persistent along the water column, growth rates of population necessarily vary within layers. For populations synchronized to the photoperiod, difference in incident irradiance with depth can induce the retardation of the cell cycle whereas nutrient stratification and pigment quenching due to high UV can also completely invert its timing between deep and surface layers (Vaulot and Marie, 1999). Water mixing could thus influence the growth rate of a patchy population, as it should homogenize the nutrient pool and light availability. The vertical gradient of cell size can lead to growth rate differences between model and mean biovolume estimates. Presence of non-vegetative larger cells (Blackburn et al., 1989), displaying smaller division probability, could explain the biovolume ratio being superior to the model ratio when mean size is used. Stratification of the water column, as demonstrated by the time series of hydrological variables recorded, should increase the differences between these two estimations. Although there is strong evidence of the inhibitory effects of turbulence on dinoflagellate cell division and growth (Berdalet, 1992), the rise of C4–C5 cluster growth rates at the sampling point with the wind was likely due to higher growth rates at the bottom, suggesting the presence of a nutricline before the mixing, and a decrease in the light limitation due to turbidity dispersal. The influence of drop of temperature on growth rates is improper with no or positive correlation in the range 15–20°C for phytoplankton (Eppley, 1972) and particularly for species of the genus *Gymnodinium* (Thomas, 1975; Nielsen, 1996; Yamamoto et al., 2002).

## Conclusion

In this study abundances and optical properties of phytoplankton community were monitored in autumn in one of the biggest European brackish lagoons using an autonomous flow cytometer especially designed for photosynthetic microorganisms. This *in situ* monitoring carried out in the Berre lagoon and remotely operated (thanks to internet connection) enabled to follow at the single cell level the dynamics of a large diversity of phytoplanktonic functional groups, as defined by flow cytometry, in response to brief and sudden environmental forcings. In October, the concentrations of 2 groups of picophytoplankton, 6 groups of nanophytoplankton, and 4 groups of microphytoplankton were measured at high frequency before, during, and after a strong wind event, providing in near real time the *in situ* structure of the phytoplankton community. Data analyses revealed a high variability of the autotrophic community in response to this natural forcing, that has been undetected by the routine survey conducted monthly by a conventional manual sampling. Phytoplankton responses to wind have also been reproduced during a second event and have been previously described in the literature, leading to the definition of the concept of response functional groups (Thyssen et al., 2014).

Besides, a potential application to harmful algal bloom monitoring is conceivable with this technology combining flow cytometry and image analysis (Campbell et al., 2010). In this study, we managed to identify red tide species and their dynamics at population and intrinsic levels. A stable hydrological environment triggered their proliferation before the wind event, resulting in a chlorophyll biomass peak, with the exception of the *Gymnodinium* cluster. The turbulent mixing generally affects the spatial distribution of phytoplankton by physical transport and may hide influence on the net abundance of populations by physiological changes that need to be measured at the individual level. To address this issue, estimates of *in situ* population growth rates have been calculated using the light scatter signal intensity recorded during the passage of each cells through the 488 nm laser beam. This signal was strongly correlated to the biovolume of the cells pictured by the “image-in-flow” device. Growth rates were calculated from the diel changes of size distribution monitored by the instrument thanks to the high frequency measurements that are difficult to obtain using more conventional methods. The increase of asexual reproductive rates was predicted by the model of Sosik et al. (2003) for the two phased clusters, probably as a result of the attenuation of light/nutrient limitation at the sampling point in response to the homogenization of the water column.

Although chlorophyll biomass dropped during the wind-induced mixing, the evolution of population growth rates suggest that in some cases, the calculation of NPP based on ocean color might not match the estimation of photosynthetic carbon fixation. Observing marine microorganisms at the single cell level with an automated flow cytometer operating at high frequency (up to several sampling and analyses per hour) takes into account the short-term variability of phytoplankton. Coupled to other hydrological sensors also operating at high frequency it should bring a new insight into the phytoplankton structure and functioning. It should also fill the gap in the primary production budget estimations based mostly on low frequency sampling (typically every month or twice a month) that cannot take into consideration pulsed events. Not taking into account the fast biomass pulses of phytoplankton could significantly impact the estimations of biogeochemical fluxes and budgets on an annual scale (Lomas et al., 2009).

### Conflict of interest statement

The authors declare that the research was conducted in the absence of any commercial or financial relationships that could be construed as a potential conflict of interest.
